# Supplementary material for: Development and Physico-Chemical Characterization of Healthy Puff Pastry Margarines Made from Olive-Pomace Oil
Source: Foods. 2022 Dec 15;11(24):4054. doi: 10.3390/foods11244054 (PMC9778141; doi:10.3390/foods11244054)
Supplement: Supplementary file 1 [file foods-11-04054-s001.zip › foods-2070708-supplementary.pdf]

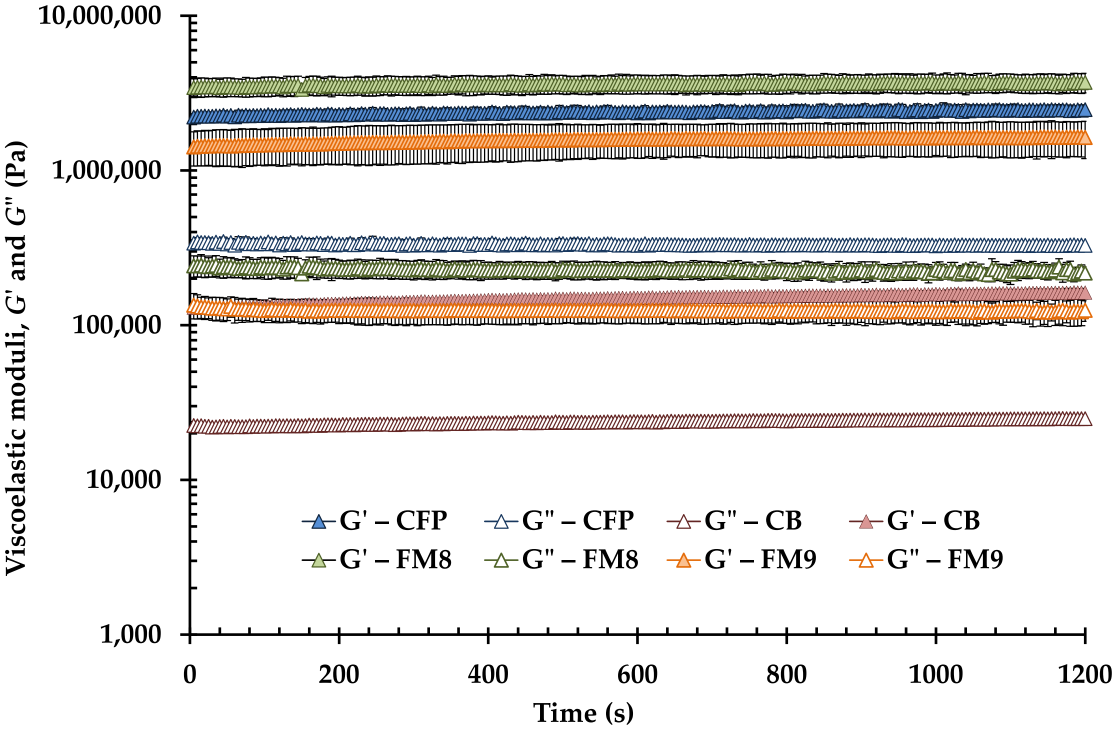

Figure S1. Time sweeps at 1 Hz and at 20 °C of a commercial fatty preparation (CFP), a commercial butter (CB) and two formulated margarines (FM8 and FM9). Shear stress (σ) was chosen in the LVR (20 Pa for CB and 200 Pa for CFP, FM8 and FM9). *G*′, elastic modulus; *G*″, viscous modulus.

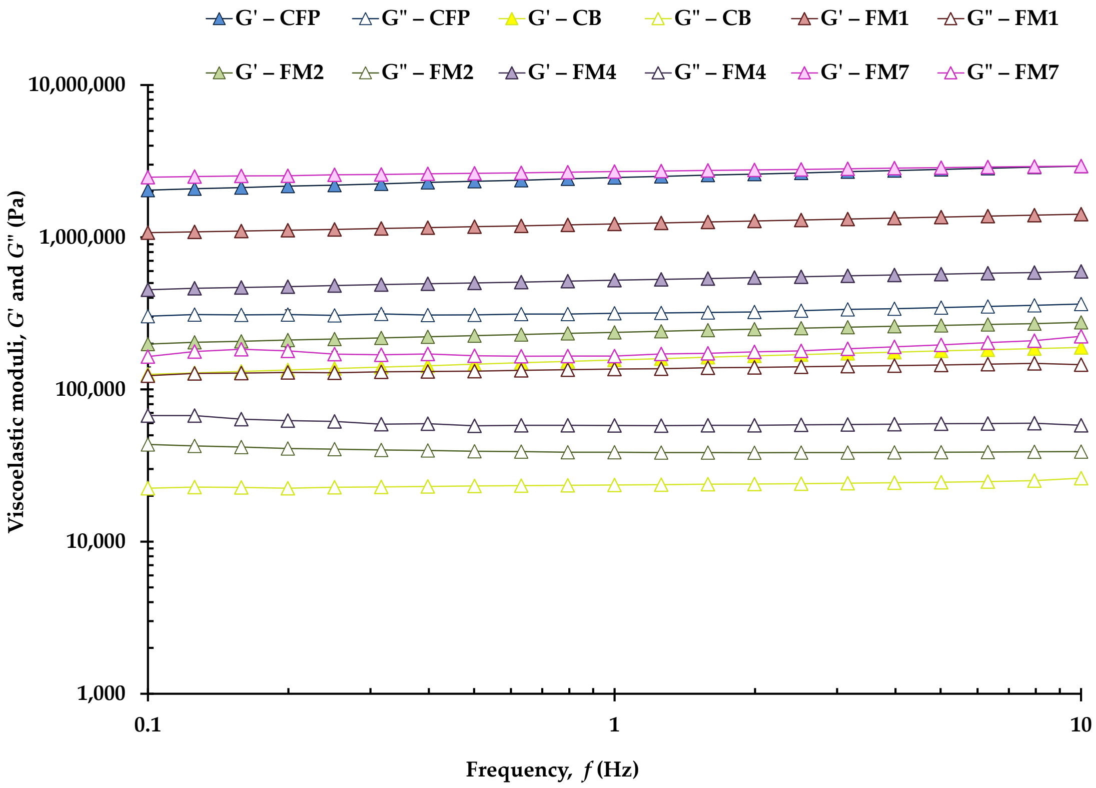

Figure S2. Frequency sweeps carried out at 1 Hz and at 20 °C to determine the mechanical spectra of a commercial fatty preparation (CFP), a commercial butter (CB) and different formulated margarines (FM1, FM2, FM4 and FM7). *G*′, elastic modulus; *G*″, viscous modulus.

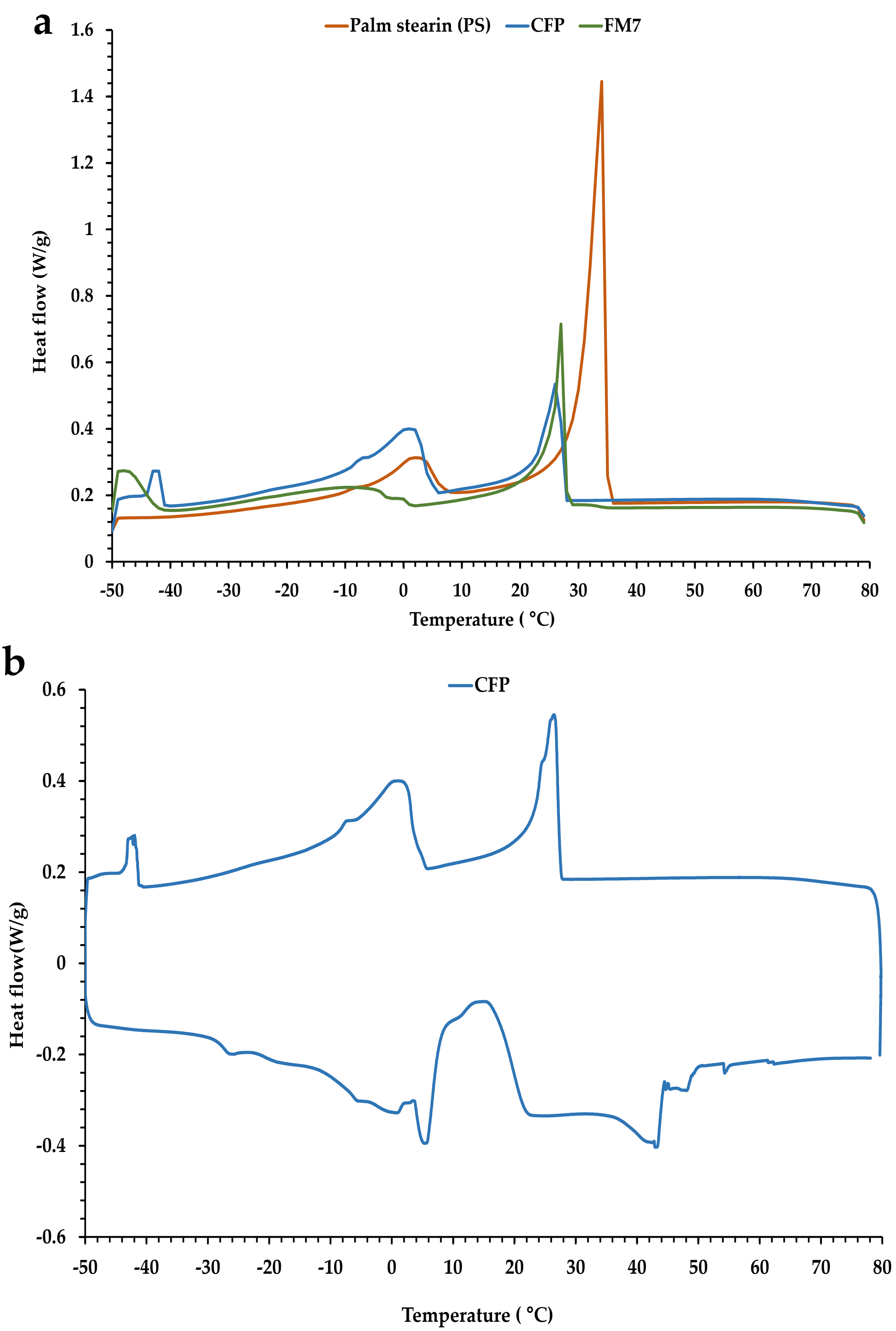

Figure S3. Thermograms obtained by DSC; (**a**) cooling crystallization profile of palm stearin (PS), a commercial fatty preparation (CFP) and one formulated margarine (FM7); (**b**) cooling crystallization and heating melting profiles of a commercial fatty preparation (CFP).

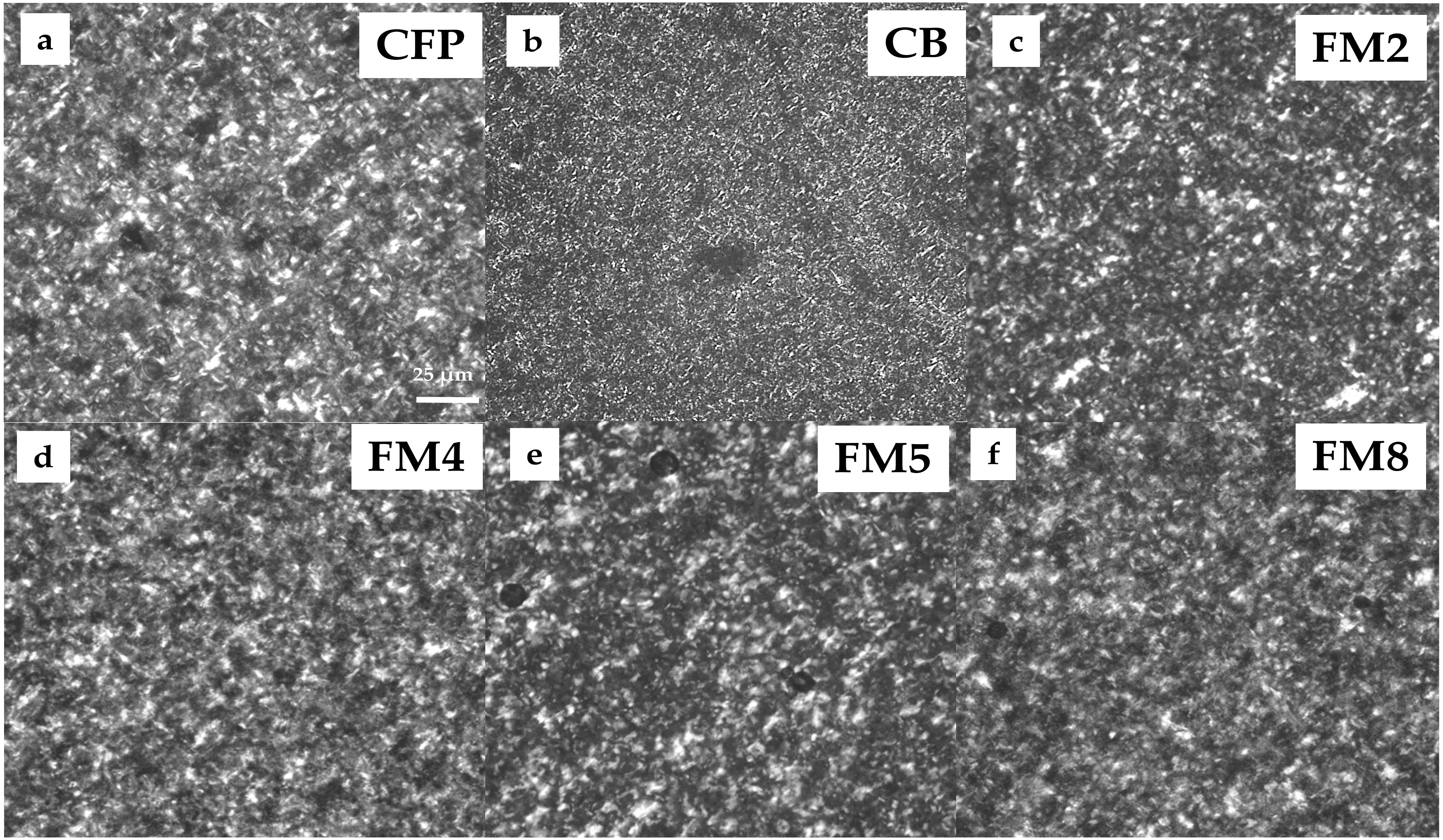

Figure S4. Polarized light microscopy (PLM) images of a commercial fatty preparation (CFP), a commercial butter (CB) and different formulated margarines (FM2, FM4, FM5 and FM8).
